# Role of Nitric Oxide in Plant Senescence

**DOI:** 10.3389/fpls.2022.851631

**Published:** 2022-04-05

**Authors:** Adil Hussain, Farooq Shah, Farman Ali, Byung-Wook Yun

**Affiliations:** ^1^Department of Entomology, Abdul Wali Khan University Mardan, Mardan, Pakistan; ^2^Department of Agronomy, Abdul Wali Khan University Mardan, Mardan, Pakistan; ^3^Department of Applied Biosciences, College of Agriculture and Life Science, Kyungpook National University, Daegu, South Korea

**Keywords:** nitric oxide, senescence, programmed cell death, ageing, ROS

## Abstract

In plants senescence is the final stage of plant growth and development that ultimately leads to death. Plants experience age-related as well as stress-induced developmental ageing. Senescence involves significant changes at the transcriptional, post-translational and metabolomic levels. Furthermore, phytohormones also play a critical role in the programmed senescence of plants. Nitric oxide (NO) is a gaseous signalling molecule that regulates a plethora of physiological processes in plants. Its role in the control of ageing and senescence has just started to be elucidated. Here, we review the role of NO in the regulation of programmed cell death, seed ageing, fruit ripening and senescence. We also discuss the role of NO in the modulation of phytohormones during senescence and the significance of NO-ROS cross-talk during programmed cell death and senescence.

## Introduction

In plants, senescence is a ubiquitous and unavoidable process of ageing that ultimately leads to death, just like all other living organisms. Plants experience both age-related and stress-induced developmental ageing. As Schippers et al. (2015), describe it as “*living to die and dying to live*,” the process of senescence is extremely important for the survival of plants. Technically, senescence is the programmed, coordinated breakdown of leaf cells under strict genetic control, as part of the overall development of plants, to remobilize essential nutrients, ensure reproductive success and provide the necessary phenotypic plasticity for adapting to the changing climate (Schippers et al., 2015). This is also why senescence is thought to be a form of programmed cell death (PCD). Senescence is associated with a gradual loss of the green colour as chloroplasts are the first cellular organelles to be broken down (Dodge, 1970). Selective chloroplast breakdown helps salvage a major portion of essential macromolecules such as proteins and lipids for recycling during the final stages (Ischebeck et al., 2006) and provides up to 80% of the final nitrogen (N) content of grains (Girondé et al., 2015). However, it is important to mention that a certain fraction of plastids may be turned into chloroplasts by degrading the photosynthetic machinery.

Nitric oxide (NO) is a gaseous signalling molecule with key roles in several physiological processes involved in plant growth and development. NO has established roles in seed germination (Sirova et al., 2011; Deng and Song, 2012; Piterkova et al., 2012; Arc et al., 2013a,b; Albertos et al., 2015), plant growth (Kopyra and Gwozdz, 2004), phytohormone perception and homeostasis (Lopez-Molina et al., 2002; Huang et al., 2004; Terrile et al., 2012; Mur et al., 2013; Lang and Zuo, 2015), flower formation, reproduction and seed setting (Kwon et al., 2012), fruit ripening (Manjunatha et al., 2012; Corpas and Palma, 2018), plant defence against infection (Kawakita et al., 2004, 2010; Malik et al., 2011; Kawakita, 2014; Yun et al., 2016; Khan et al., 2019) and several abiotic stresses (Mun et al., 2017; Khan et al., 2019; Nabi et al., 2019). The free radical NO reacts with various extracellular/intracellular macromolecules forming a series of other molecules including peroxynitrite (ONOO^−^), nitrosonium ions (NO+), NO radicals (NO), higher nitrogen oxides (NOx), iron dinitrosyl complexes, and *S*-nitrosothiols (SNOs) among others. These molecules are collectively called reactive nitrogen species (RNS; Di Stasi et al., 2002). The process of S-nitrosylation, the covalent binding of an NO moiety to a reactive cysteine thiol to form an SNO, is the post-translational modification (PTM) of proteins that has emerged as a key redox-based PTM of proteins in plants. An armada of proteins involved in key physiological processes has been shown to be targets of S-nitrosylation in plants and animals. The direct role of NO in plant senescence has also been briefly described in a few studies (Corpas and Palma, 2018; Bruand and Meilhoc, 2019) such as leaf (De Michele et al., 2009), cotyledon (Du et al., 2013) and stress-induces senescence (Terrón-Camero et al., 2019). In plants, NO is produced through several different reductive and oxidative, enzymatic and non-enzymatic pathways (Hussain et al., 2022). Several studies hint towards NO production in the chloroplasts and mitochondria (Galatro et al., 2013; Vanlerberghe, 2013) which are the first cellular organelles to be dismantled during senescence (reviewed by León and Costa-Broseta, 2020). In animal systems, NO has a well-established regulatory role in apoptosis and cell death (Kim et al., 1999, 2001; Brüne, 2003; Snyder et al., 2009), a process analogous to senescence in plants. Interestingly, NO acts as a signalling molecule or a toxic molecule depending upon the situation.

## Nitric Oxide Signalling During Programmed Cell Death

**Figure 1:**
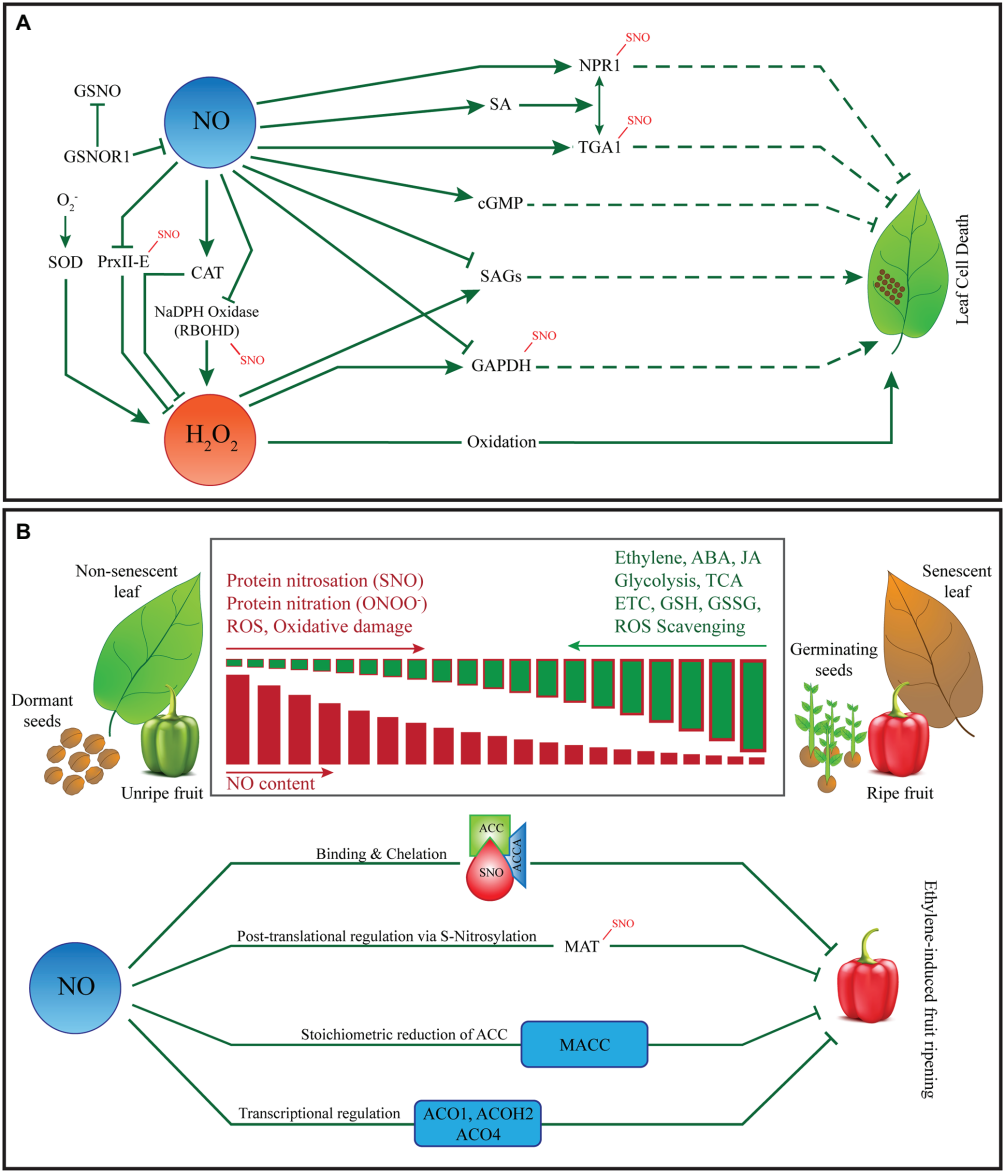
Role of nitric oxide in fruit ripening, programmed cell death and leaf senescence. **(A)** NO and ROS-mediated control of programmed cell death. Plants rapidly-produce NO and various ROS in response to biotic and/or abiotic stresses leading to intrinsic execution of the target cells. NO and ROS can trigger PCD either synergistically or independently. Both NO and ROS are implicated in the hypersensitive response (HR), characterized by the execution of the infected and surrounding cells, is a well-known form of PCD in plants. However, significant cross-talk exists between NO and ROS during the establishment of HR. The establishment of HR warrants a critical balance between intracellular levels of NO and ROS. Several key proteins such as non-expressor of pathogensi-related genes 1 (NPR1), TGA1 (transcriptional activators that specifically bind to 5′-TGACG-3′), glyceraldehyde-3-phosphate dehydrorgenase (GAPDH), respiratory burst oxidase homolog D (RBOHD) are involved in HR are known to be S-nitrosated by NO or affected by the NO-ROS cross talk. NO is also known to scavenge H_2_O_2_ under certain circumstances thereby protecting plant cells from ROS-mediated damage. **(B)** NO-mediated regulation of senescence, seed dormancy and fruit ripening. NO affects leaf senescence by modulating phytohormones such as ABA, ET, and JA. Though the role of NO in seed ageing is not well-defined, NO is known to break seed dormancy *via* stimulation of ET production. NO donors and arginine (Arg) abolishes seed dormancy and stimulates germination whereas, the application of NO scavengers (such as cPTIO, L-Name, and CAN) promotes seed dormancy. NO-mediated removal of seed dormancy and promotion of germination is associated with alternations of nitrated and biotinylated proteins, ABA, JA and protein nitration. NO has also been implicated in fruit ripening. As the fruit ripens, ROS and RNS anabolic vs. catabolic rates change significantly altering the overall nitro-oxidative environment at the cellular level ultimately affecting the NO-dependent S-Nitorsation and Tyrosine Nitration events in fruit. Furthermore, NO mediated binding and chelation, S-nitrosylation of methionine adenosyl transferase (MAT), stoichiometric reduction of ACC and transcriptional regulation of several genes involved in the ethylene pathway plays an important role in fruit ripening.

In plants, programmed cell death is an important process regulating several aspects of plant growth and development. Besides, it is a natural mechanism in place to execute infected cells during plant responses to biotic and abiotic stresses. PCD is mediated by NO and reactive oxygen species (ROS; Wang et al., 2013b; Figure 1A). Plants rapidly-produce NO and various ROS in response to biotic and/or abiotic stresses leading to the intrinsic execution of the target cells. Interestingly, these small redox-active molecules can trigger PCD either synergistically or independently. In plants, a well-known form of PCD is the hypersensitive response (HR) characterized by the rapid execution of the infected and surrounding cells. Some HR characteristics in plants are similar to apoptosis in animal cells such as DNA cleavage, chromatin condensation, cytoplasmic vacuolization and membrane dysfunction (Greenberg and Yao, 2004; Choi et al., 2013; Iakimova et al., 2013), yet there are key differences between the two processes. For example, plant HR is usually accompanied by necrosis of infected and surrounding plant cells, a key mechanism for containing the pathogen within the necrotic cells and restricting its further spread to the healthy tissues. Furthermore, recent literature indicates that certain types of plant HR appears similar to the iron dependent cell death known as ferroptosis; which is also characterized by the accumulation of lipid peroxides (Yang and Stockwell, 2016). Recent experiments by (Dangol et al., 2019) indicate that iron- and ROS-dependent signalling cascades converge to regulate ferroptosis and HR in rice after avirulent *Magnaporthe oryzae* infection suggesting that HR and ferroptosis may share the same molecular machinery under certain conditions. In animal system, the oxidoreductase (cytochrome P450 oxidoreductase) contributes to phospholipid peroxidation during ferroptosis (Zou et al., 2021). Yan et al. (2021) showed that coupling of cytochrome P450 oxidoreductase and cytochrome B5 reductase 1 (CYB5R1) mediates the production of H_2_O_2_, which subsequently drives lipid peroxidation and ferroptosis through the iron-catalysed Fenton reaction. Although significant cross-talk exists between NO and ROS, both are implicated in HR. However, it is clear that a balance between intracellular levels of NO and ROS is a critical determinant for HR (Delledonne et al., 2001). Pathogen attack is followed by a rapid burst of NO and a “biphasic” production of ROS at the site of infection (Romero-Puertas et al., 2004) indicating the NO and ROS act synergistically or at least in combination to promote HR. However, NO and ROS can independently release cytochrome *c* from mitochondria, affecting the caspase-like signalling pathway that leads to HR (Mur et al., 2006). The mitogen-activated protein kinases (MAPKs) and phosphatases are key components of plant defence that are affected by NO and ROS subsequently affecting the establishment of HR. These are examples of cases where NO and ROS appear to converge promoting HR in response to infection. As described above, a major route for NO bioactivity is *via* the canonical PTM of proteins known as S-nitrosylation, several key proteins involved in HR are known to be S-nitrosated by NO or affected by the NO-ROS cross talk. For example, ROS signalling in plants is mediated by GAPDH is a direct target of NO-mediated S-nitrosylation that blunts its activity (Lindermayr et al., 2005). Furthermore, Peroxiredoxin-2E (PrxIIE) and Methionine adenosyl transferase (MAT) are other examples of key proteins involved in HR that are affected by ROS and NO-mediated S-nitrosylation (Dietz, 2003; Horling et al., 2003; Romero-Puertas et al., 2007a,b). However, NO is also known to scavenge H_2_O_2_ under certain circumstances thereby protecting plant cells from ROS-mediated damage (Beligni et al., 2002; Guo and Crawford, 2005). In plants, the enzyme S-nitrosoglutathione reductase 1 (GSNOR 1) regulates the global levels of S-nitrosated proteins (Hussain et al., 2019). NO S-Nitrosylates NADPH oxidase Respiratory burst oxidase homolog D (AtRBOHD) at Cys890 which blunts its function in ROS synthesis (Yun et al., 2011; Figure 1). Several studies involving loss of function *gsnor* mutant plants indicate that GNSOR1 regulates SA signalling, thermotolerance and acts downstream of superoxide to regulate cell death (Feechan et al., 2005; Lee et al., 2008; Chen et al., 2009; Wang et al., 2010).

In plants, the Class II (CGFS-type) glutaredoxins (GRXs) act as FeS cluster transferases. On the other hand, the Class I (CxxC/S-type) GRXs act as oxidoreductases and participate in oxidative signalling. GRx and FeS binding involves active site cysteines and glutathione (GSH; Couturier et al., 2015; Trnka et al., 2020). In eukaryotic cells, the CGFS-type GRXs function in iron metabolism, i.e., the biogenesis of plastidal, mitochondrial, or bacterial FeS clusters (Mühlenhoff et al., 2003; Moseler et al., 2015) and iron trafficking to the cytosol. Since these FeS-transfering GRXs are redox-active, they are also affected by NO and ROS. An important hub through which plants regulate PCD is through the involvement of ROS and NO in iron homeostasis. Iron is an essential micronutrient for plants. Iron in the form of heme and Fe-sulfur (FeS) clusters is required for primary metabolic processes, such as respiration, cell proliferation and differentiation, DNA biosynthesis and repair, photosynthesis and biosynthesis of chlorophyll and hormones (Camprubi et al., 2017). Despite its importance as an essential micronutrient, iron overload can prove to be toxic due to its potent electro-chemistry. Iron reacts with ROS such as H_2_O_2_ *via* the Fenton reaction; a catalytic reaction through which iron converts H_2_O_2_ into biologically more toxic ROS including the hydroxide radical (HO; Winterbourn, 1995). These potent ROS result in oxidative damage of nucleic acids, lipids and proteins. In severe cases the damage manifests in the form of PCD. The production of hydroxyl radical has been reported *via* the Fenton reaction in submitochondrial particles under oxidative stress (Thomas et al., 2009). On the other hand, NO appears to up-regulated Fe uptake by the plants and increase its bioavailability for metabolic functions. The formation of NO-Fe complexes is considered as one of the strategies for storing and stabilizing free NO and Fe within the cells (Hsiao et al., 2019). NO also plays a key role in the modulation of ferritin; the cellular Fe store house. Ferritin can store about 2,000–4,500 Fe atoms. Loss of function ferritin triple mutant plants *fer1,3,4* exhibit massive Fe accumulation (Roschzttardtz et al., 2013). As described above, physiological excess of Fe results in oxidative damage due to the formation of hydorxyl radical *via* the Fenton reaction. NO is required for Fe-induced ferritin synthesis. NO acts downstream of Fe in the induction of ferritin transcripts and is a key signalling molecule for the regulation of Fe homeostasis in plants (Murgia et al., 2002; Tewari et al., 2020).

## NO and ROS Cross-Talk During Senescence

As described above, senescence is thought to be just another form of plant PCD. It is the final stage of plant development that occurs under strict genetic control but is also triggered and affected by environmental conditions. Furthermore, phytohormones such as abscisic acid (ABA), Auxin (IAA), cytokinin (CK), ethylene (ET), jasmonic acid (JA) and salicylic acid (SA) also affect the process of senescence (Lim et al., 2007). In plants, senescence is characterized by a dramatic increase in the H_2_O_2_ content in leaf tissues which results in the oxidation of macromolecules, proteins and lipids and triggers the expression of genes involved in senescence (Cui et al., 2013) indicating a dual role of ROS during senescence. Balazadeh et al. in 2010 and 2011 reported rapid and strong induction of senescence-associated NAC genes (sen-NACs) by H_2_O_2_ in roots and leaves (Balazadeh et al., 2010, 2011). Similarly, the WRKY253 and WRKY53 genes have been described to regulate plant senescence in a redox-dependent manner (Kim et al., 2009, 2017; Doll et al., 2020). ROS and NO induce senescence of rudimentary leaves and the expression profiles of the related genes in Litchi (*Litchi chinensis*) plants (Yang et al., 2018). However, significant cross-talk exists between various RNS and ROS during senescence and PCD (Figure 1A). On the other hand, both pro-and anti- senescence roles have been described for NO, depending upon the situation such as its subcellular location and concentration. NO is known to scavenge H_2_O_2_ and alleviate ROS toxicity thereby delaying senescence (Sang et al., 2008). Loss of function mutant plants *nos1/noa1* which are NO-deficient, exhibit early senescence (Niu and Guo, 2012). Furthermore, ectopic expression of the NO-degrading dioxygenase (NOD) enzyme in plants results in early senescence (Mishina et al., 2007). Plants lacking the plasma membrane-localized cyclic nucleotide-gated ion channel 2 (CNGC2) exhibit an early senescence phenotype. For example, the *Arabidopsis thaliana* CNGC2 loss of function *dnd1* plants exhibits loss of chlorophyll, H_2_O_2_ production, tissue necrosis and lipid peroxidation with significantly lower levels of NO as compared to wild type (WT) plants (Ma et al., 2010) indicating a contrasting behaviour of ROS and NO during senescence. This protective role of NO against ROS-mediated senescence can be associated with the activity of the antioxidant machinery that negatively regulates chlorophyll break down and promotes the stability of the thylakoid membranes during senescence (Liu and Guo, 2013).

Case studies where NO plays a pro-senescence role have also been reported. The rice (*Oryza sativa*) nitric oxide excess 1 (NOE1) gene encodes a rice catalase enzyme (OsCATC). The rice NO-accrual mutant *noe1* accumulates higher amounts of H_2_O_2_ in the leaves that consequently promotes NO production *via* the activation of the nitrate reductase (NR) enzyme. Removal of excess NO resulted in reduced cell death, indicating the NO acts as a mediator of H_2_O_2_-induced cell death in this case. In addition, further reduction of cellular SNO levels by the over-expression of rice GSNOR reduced cell death in *noe1* rice plants. In another study, exogenous application of NO led to rapid production of H_2_O_2_ and induction of cell death in maize (*Zea mays*) leaves (Kong et al., 2013). These results indicate that NO and NO-mediated S-nitrosylation of proteins are important mediators of H_2_O_2_ mediated cell death and senescence (Lin et al., 2011; Wang et al., 2013a).

## NO-Mediated Control of Senescence *via* Modulation of Phytohormones

Phytohormones are chemical messengers that coordinate various physiological activities in plants. The various types of phytohormones include IAA, gibberellins (GAs), CK, ET, ABA, SA, JA, and brassinosteriods (BR). For plants, life is not possible without these messenger molecules as they support key physiological events throughout plant life. Significant progress has been made in understanding the underlying mechanisms of phytohormone perception, biosynthesis, transportation, signal transduction, and downstream effects. An interesting feature is that the signalling pathways of some phytohormones work in parallel (synergistic relationship) whereas, those of others intersect each other (antagonistic relationship). Normal plant physiology requires basal levels of all these phytohormones. However, under stress conditions, drastic changes in the phytohormonal pathways are a key feature of plant responses to these stresses.

NO-mediated control of phytohormones is well-known. NO affects leaf senescence by modulating the activity of phytohormones such as ABA, ET, and JA (Figure 1B). NO has been shown to frequently interact with phytohormones leading to remarkably complex signalling cascades. Accumulating evidence in the literature indicates the interaction of NO with virtually all major phytohormones affecting their biosynthesis, perception, metabolism, transport, and downstream effects (Freschi, 2013). Furthermore, the type and magnitude of the interaction may vary under different conditions. So far key roles for NO have been described in the regulation of ABA, ET, IAA, GAs, JA, SA and BR in several plant species both at the transcriptome (Hussain et al., 2016) as well as the proteome level. NO-mediated regulation of senescence has been reported in several studies. The exogenous application of NO donors extends the post-harvest life of fruits and vegetables and slows down flower maturation (Leshem and Haramaty, 1996). NO has also been reported to counteract leaf senescence caused by ABA and methyl jasmonate (MeJA) in rice (Hung and Kao, 2004). NO concentration increases to delay MeJA-induced leaf senescence in plants after treatment with a low concentration of SA (Ji et al., 2016). The role of CK in fruit maturation and senescence is extremely important. Application of exogenous CK simultaneously stimulates the expression of the nitrate reductase (NR) enzyme for the production of NO and delays senescence (Magalhaes et al., 2000; Tun et al., 2001; Planchet et al., 2006; Kolbert et al., 2019). The CK signalling pathway is mediated by a phosphorelay system that sequentially transfers phosphoryl groups from the CK receptors to histidine phosphotransfer proteins (AHPs) and response regulators (ARRs). Feng et al. (2013) described that the S-nitrosylation of AHP1 at Cys 115 represses its phosphorylation and the subsequent transfer of the phosphoryl group to ARR1. They also showed that a non-nitrosatable mutant AHP1 is insensitive to NO in repressing its phosphorylation relieving the inhibitory effect of NO. These findings illustrate NO-mediated control of CK signalling during plant growth and development. CK also interacts with ET which is another important phytohormone required for senescence. Wilhelmova et al. (2006) reported that tobacco plants with high or low levels of CK show perturbed levels of ET and NO during senescence as compared to plants with normal levels of CK. They also reported that NO and ET production was under the control of genes other than those regulating senescence. Although their results did not indicate a direct link between ET production and CK levels, they recorded low NO levels. Furthermore, they also recorded that low CK level was associated with increased NO production. From the above studies, the interaction of NO and CK appears to be dose-dependent. Application of NO donors, [N-tert-butyl-α-phenylnitrone (PBN), sodium nitroprusside (SNP), 3-morpholinosydonimine, and AsA + NaNO_2_] has been reported to inhibit the increase of H_2_O_2_ levels during ABA- and JA-induced senescence in rice (Hung and Kao, 2004). The NO deficient mutant *nos1/noa1* exhibits early senescence. On the other hand, the ethylene insensitive 2 (EIN2) positively regulates ET-induced senescence. Niu and Guo (2012) characterized the *ein2-1nos1/noa1* double mutant plants to determine the relationship of NO and ET during senescence. Their results indicated that the dark-induced early senescence phenotype of *nos1/noa1* was suppressed by the EIN2 mutation *via* a reduction in chlorophyll degradation, improving thylakoid membrane stability and significant inhibition in the up-regulation of senescence-associated genes, suggesting that EIN2 is involved in NO signalling during senescence. On the other hand, NO deficiency accelerates chlorophyll degradation and stability of the thylakoid membrane during dark-induced leaf senescence (Liu and Guo, 2013).

## Reactive Nitrogen Species and Ageing in Seeds

Ageing can simply be defined as growing old. Ageing means going through all phases of growth and development from germination to growth, senescence and seed production. Plants go through both stress-induced and age-related developmental ageing. Seed performance is a major determinant of crop yield and a base for plant growth and development. The longevity of seeds varies among the different plant species. However, seed quality and vigour deteriorates with age and is affected by internal and external factors (Sano et al., 2015).

Seed germination performance is a major determinant of crop yield. Deterioration of seed quality with age is associated with the accumulation of DNA damage. Ageing in seeds is characterized by a loss of membrane stability at the cellular level, nucleic acid degradation, an increase in oxidative damage due to ROS accumulation and a concomitant reduction in the antioxidant potential (Bailly et al., 2008; El-Maarouf-Bouteau et al., 2011). Though the role of NO in seed ageing is not well-defined, NO is known to break seed dormancy *via* stimulation of ET production (Gniazdowska et al., 2007, 2010). Furthermore, the application of NO donors and arginine (Arg) to apple (*Malus domestica*) seeds abolishes the dormancy of the embryo and stimulates germination (Krasuska et al., 2016). On the other hand, the application of NO scavengers (cPTIO, L-Name, CAN) promotes seed dormancy. They also reported that NO-mediated removal of seed dormancy and promotion of germination is associated with alternations of nitrated and biotinylated proteins (Krasuska et al., 2016). This NO-mediated removal of apple seed dormancy has been linked to differential expression of genes associated with ABA, JA and RNA nitration (Andryka-Dudek et al., 2019). Several other studies have described the importance of NO for the transition of seeds from dormancy to germination (Bykova et al., 2015; Signorelli and Considine, 2018; Figure 1B). NO treatment is regularly used for seed stratification, accelerating germination and improvement of seed vigour. At the molecular level, NO is involved in S-nitrosylation and tyrosine nitration of different proteins as well as nitration of nucleic acids and fatty acids (Mata-Pérez et al., 2017; Arasimowicz-Jelonek and Floryszak-Wieczorek, 2019). NO enhances the desiccation tolerance of recalcitrant *Antiaris toxicaria* seeds *via* S-nitrosylation (Bai et al., 2011) and reduces the accumulation of H_2_O_2_ (Bai et al., 2011). NO has been reported to act as an H_2_O_2_ scavenger in germinating wheat (*Triticum aestivum*) seeds (Cakmak et al., 1993). NO delays ageing of soybean (*Glycine max*) cotyledons *via* chlorophyll stabilization (Jasid et al., 2006).

As described, deterioration in seed vigour and low germination are the characteristics of seed ageing. Controlled deterioration treatments (CDT) are often used by researchers under laboratory conditions to investigate the effects of ageing on seeds. CDTs often involve gradual changes in storage temperature and humidity conditions. In 2018, He et al. (2018) investigated the effects of CDT-induced ageing on elm seeds and reported a significant reduction in seed vigour with a concomitant reduction in cellular NO levels. However, treatment of the seeds with the NO donor SNP before CDT treatment promoted the overall vigour of the seeds with a significant increase in their germination. Using the biotin switch method, they identified 82 putative S-nitrosated proteins involved in carbohydrate metabolism. In addition, they also reported 163 metabolites that responded to both NO and the CDT. Similarly, Mao et al. (2018) reported NO-mediated regulation of seedling growth and mitochondrial responses in aged oat seeds. They reported a significant increase in H_2_O_2_ accumulation and loss of vigour in artificially aged oat seeds. However, SNP treatment of aged seeds protected seeds from H_2_O_2_-meidated damage and improved seed vigour *via* enhancement of ROS scavenging in the mitochondria *via* upregulation of catalase (CAT), dehydroascorbate reductase (DHAR), glutathione reductase (GR), monodehydroascorbate reductase (MDHAR), activity.

## Nitric Oxide in Fruit Ripening

The development of different plant parts such as fruits can be broadly divided into three major stages in sequence; growth, maturation, and senescence. The *growth* period can be characterized by cell division and enlargement, which accounts for the increase in the overall biomass or size of the fruits. The *maturation* stage is reached just before the end of growth and involves the development of colour, odour and taste or flavour. Senescence starts when the overall magnitude of degradative processes is more than the biosynthetic processes. Fruit ripening and senescence involve highly coordinated complex biochemical changes at the cellular level involving transcriptional changes in ripening-associated genes (RAGs), transcription promoters and repressors, enzymes and metabolic rewiring (Fuentes et al., 2019). In general, climacteric fruits ripen after harvest whereas, non-climacteric fruits do not ripen after harvest. Bananas (*Musa* spp.) left on a kitchen table will ripen because they are climacteric and continue to produce ET even after being harvested. However, strawberries (*Fragaria ananassa*) do not ripen after harvest as they do not produce ET after being harvested.

Though ET plays a key role in fruit ripening, NO has also been implicated in fruit ripening and its role in regulating the ripening associated genetic and metabolic networks has just started to be unravelled. NO has emerged as a promising molecule for extending the post-harvest life of fruits without affecting the quality traits such as colour, texture, aroma and taste. In this regard, the initial works of Leshem’s group (Leshem et al., 1998, 2001; Leshem and Pinchasov, 2000) are of particular importance. Since then, NO has emerged as an important RNS involved in the maintenance of agronomic traits in fruits and the regulation of physiological processes at the molecular level. As the fruit ripens, ROS and RNS anabolic vs. catabolic rates change significantly altering the overall nitro-oxidative environment at the cellular level ultimately affecting the NO-dependent S-nitrosylation and tyrosine nitration events in fruit. At the transcriptional level NO regulates the expression of RAGs and those involved in fruit antioxidant defence, whereas at the post-translational level, antioxidant enzymes have been identified as targets of both protein nitration and S-nitrosylation in fruits (Chaki et al., 2015; Corpas and Palma, 2018; Gramegna et al., 2019). In addition, NO is also involved in metabolic rewiring to regulate fruit ripening (Gramegna et al., 2019; Zuccarelli et al., 2020).

Several studies have reported the negative effects of NO on ET biosynthesis in fruit thereby regulating fruit ripening (Manjunatha et al., 2012) in both climacteric fruits such as banana (Cheng et al., 2009), tomato (*Solanum lycopersicum*; Eum et al., 2009), apple (Rudell and Mattheis, 2006) and pepper (*Capsicum annum*; Chaki et al., 2015) as well as non-climacteric fruits such as strawberries (Zhu and Zhou, 2007). NO affects ET-induced ripening *via* transcriptional regulation of 1-carboxylic acid oxidase (ACO) genes ACO1, ACOH2 and ACO4; S-nitrosylation of methionine adenosyl transferase (MAT) and stoichiometric reduction of ACC (a precursor of ethylene) into 1-malonyl aminocyclopropane-1-carboxylic acid (MACC). NO also inhibits ET-induced fruit ripening *via* binding and chelation to form an ACC-ACC oxidase–NO (Figure 1B). Furthermore, there are several unidentified and proposed models for NO-mediated suppression of ET-induced fruit ripening.

Functional genomic studies of the tomato *shr* mutant plants overproducing NO indicated that higher concentrations of NO shift metabolic profiles and suppresses fruit growth and ripening (Bodanapu et al., 2016). Zuccarelli et al. (2020) showed that pre-climacteric exogenous application of NO in tomato delays ripening. Through further transcriptomic analysis, they showed that approximately one-third of the fruit transcriptome was changed as a result of NO exposure including the down-regulation of RAGs thereby restricting the production of ET and the ET sensitivity of the fruit tissues. In addition, they also recorded NO-mediated suppression of H_2_O_2_ scavenging enzymes resulting in nitro-oxidative stress and S-nitrosylation throughout the ripening process. Metabolomic analysis indicated alterations in carotenoid, tocopherol, flavonoid and ascorbate levels in the fruits following exposure to NO that ultimately affected lycopene production. However, NO had little effect on food quality parameters such as taste and aroma indicating the importance of NO from an agronomic point of view.

## Conclusion

NO plays a key role in plant biology. It regulates various physiological, biochemical and molecular aspects of senescence in plants. In fruits, NO has long been described as an anti-senescence molecule as its application delays ripening and senescence and extends the post-harvest shelf life of fruits and vegetables. NO deficient mutant plants show early or enhanced senescence phenotypes. NO counteracts leaf senescence by suppressing chlorophyll degradation and promoting thylakoid membrane stability. However, NO acts as a Pro-senescence as well as an anti-senescence agent under different circumstances. Furthermore, the production of different RNS and ROS is a routine requirement for the maintenance of normal plant physiology. During incompatible plant pathogen interactions, the plant leucin-rich repeat (LRR) receptors recognize pathogen effector molecules which subsequently induces rapid HR and cell death. Plant NO is a well-recognized player in such plant pathogen interactions. A wealth of knowledge exists indicating significant crosstalk exists between RNS and ROS although this relationship remains elusive during programmed cell death. NO and/or ROS dependent and glutathione-dependent antioxidant defence and the iron-dependent (ferroptosis) cell death are hallmarks of the plant immune system. NO appears to play critical role in regulating these processes though the exact molecular mechanisms are still being investigated. ROS burst, increase of intracellular iron and α-glutamylcysteine synthetase are considered as key markers for ferroptotic cell death in plants (Hiruma et al., 2013). NO interacts with various ROS, haem-containing proteins and glutathione within the cell. These interactions represent different avenues for NO-mediated control of cell death in plants. The role of NO in regulating programmed cell death, seed ageing, fruit ripening and overall plant senescence has just begun to be unravelled and a complete list of signalling pathways regulated by NO is still being developed and several key questions remain to be answered. What are the molecular mechanisms responsible for deciding Pro- or anti-senescence roles of NO? How NO and ROS cross talk regulates senescence and PCD? What is the role of NO in regulating PCD during incompatible plant pathogen interactions? What are the effects of NO on various phytohormones during senescence? Thus, further work is required to understand how this key molecule triggers the onset of senescence in plants.

## Author Contributions

AH: compiled the data, wrote the manuscript and made the figure. FS and FA: collected data and research papers. B-WY: wrote and reviewed the manuscript. All authors contributed to the article and approved the submitted version.

## Funding

This work was supported by Basic Science Research Program through the National Research Foundation of Korea (NRF) funded by the Ministry of Education (Grant number 2020R1I1A3073247), Republic of Korea, and a project to train professional personnel in biological materials by the Ministry of Environment.

## Conflict of Interest

The authors declare that the research was conducted in the absence of any commercial or financial relationships that could be construed as a potential conflict of interest.

## Publisher’s Note

All claims expressed in this article are solely those of the authors and do not necessarily represent those of their affiliated organizations, or those of the publisher, the editors and the reviewers. Any product that may be evaluated in this article, or claim that may be made by its manufacturer, is not guaranteed or endorsed by the publisher.
